# Study on Asphalt Pavement Surface Texture Degradation Using 3-D Image Processing Techniques and Entropy Theory

**DOI:** 10.3390/e21020208

**Published:** 2019-02-21

**Authors:** Yinghao Miao, Jiaqi Wu, Yue Hou, Linbing Wang, Weixiao Yu, Sudi Wang

**Affiliations:** 1 Beijing Key Laboratory of Traffic Engineering, Beijing University of Technology, 100 Pingleyuan, Chaoyang District, Beijing 100124, China; 2 National Center for Materials Service Safety, University of Science and Technology Beijing, 30 Xueyuan Road, Haidian District, Beijing 100083, China; 3 The Charles E. Via, Jr. Department of Civil & Environmental Engineering, Virginia Polytechnic Institute and State University, Blacksburg, VA 24061, USA

**Keywords:** pavement, macrotexture, 3-D digital imaging, entropy, decay trend

## Abstract

Surface texture is a very important factor affecting the anti-skid performance of pavements. In this paper, entropy theory is introduced to study the decay behavior of the three-dimensional macrotexture and microtexture of road surfaces in service based on the field test data collected over more than 2 years. Entropy is found to be feasible for evaluating the three-dimensional macrotexture and microtexture of an asphalt pavement surface. The complexity of the texture increases with the increase of entropy. Under the polishing action of the vehicle load, the entropy of the surface texture decreases gradually. The three-dimensional macrotexture decay characteristics of asphalt pavement surfaces are significantly different for different mixture designs. The macrotexture decay performance of asphalt pavement can be improved by designing appropriate mixtures. Compared with the traditional macrotexture parameter Mean Texture Depth (MTD) index, entropy contains more physical information and has a better correlation with the pavement anti-skid performance index. It has significant advantages in describing the relationship between macrotexture characteristics and the anti-skid performance of asphalt pavement.

## 1. Introduction

Surface texture is a very important factor affecting the anti-skid performance of pavements [1,2,3]. Due to the mutual interactions between tire and pavements during driving, the surface texture wears continuously. Some observations show that anti-skid performance decreases under the vehicle load [2,3]. The study of texture and wear characteristics are therefore helpful for civil engineers to better understand the anti-skid performance of pavements. Generally, according to different influences on the anti-skid performance, the road surface texture is divided into the macrotexture (wavelength of 0.5 to 50 mm and peak-to-peak amplitude of 0.2 to 10 mm) and the microtexture (wavelength of 0 to 0.5 mm and peak-to-peak amplitude of 0 to 0.2 mm) [4]. The Mean Texture Depth (MTD) and Mean Profile Depth (MPD) are commonly used in engineering practice to evaluate the macrotexture [5,6]. However, these indexes still need to be improved in terms of reflecting the effects of texture on anti-skid performance [7,8]. Since it is difficult to test microtextrue on the road surface, it is not required to evaluate it in engineering practices, which is mainly controlled in the stage of aggregate selection [9,10].

The development of three-dimensional testing technology provides a new method for the evaluation of pavement surface texture, as indicated in the previous study [7], like the indoor laser profiler [11], X-ray computerized tomography (CT) [12], laser technology [13], optical three-dimensional scanner [14], three-dimensional laser device [15], and four-source photometric stereo technique [16,17]. With the continuous progress of three-dimensional testing technology, many commercial three-dimensional laser scanners have been developed and applied in the measurement of the three-dimensional texture of pavement surfaces [18,19,20,21], and the corresponding resolution is gradually improving. At the same time, in order to meet the needs of rapid testing, researchers are also working to develop some on-board three-dimensional testing devices for on-site road surface texture detection [22,23].

With the fast development of three-dimensional texture testing technology for pavement surfaces, the study of fine texture features based on three-dimensional data is also carried out, like in Fourier analysis [12], fractal theory [7] and texture analysis methods in image processing [24,25]. In recent years, Shannon’s Entropy theory has been widely used as a powerful tool for image analysis [26,27,28], being significantly convenient for describing the complexity of texture. It should be noted that pavement macrotexture has been analyzed using fractal theory [7], Co-occurrence Matrix [24], gray tone difference matrix [25], and degradation analysis [29] in previous research. The introduction of entropy theory to this area can still help civil engineers better study the decay behavior of three-dimensional macrotexture and microtexture of road surfaces. There have been lots of studies on the bulk properties of pavements using various numerical and testing approaches [30,31,32,33], and the mechanism of the surface properties, e.g., pavement texture is still not fully understood. In this study, the current research progress and methods of 3D texture data acquisition in the field are introduced first. Following this, the feasibility of entropy theory in describing three-dimensional macrotexture and microtexture features is investigated. Third, the entropy of the three-dimensional texture is taken as an index to investigate the decay behavior of the macrotexture and microtexture of pavement surfaces. Finally, the advantages of three-dimensional macrotexture entropy in describing the decay of pavement anti-skid performance compared with a traditional MTD index are presented.

## 2. Field Data Collection

In order to study the decay characteristics of asphalt pavement surface texture with traffic wear, different types of asphalt surface on several highway and urban roads in Beijing were tested from November 2010 to November 2012. Seven tests were carried out during the period, where six different types of asphalt pavements were covered, including dense asphalt concrete (DAC), stone matrix asphalt (SMA), rubber asphalt concrete (RAC), ultra-thin wearing course (UTWC), micro-surfacing (MS), and open graded friction course (OGFC). The basic information of the test was described in detail in [7]. Due to the influences of pavement maintenance during the test period, the MS and OGFC measurement points did not result in continuous test data, and some DAC measurement points did not include a decay analysis due to the low traffic volume. The detailed decay analysis of the measurement points and traffic volume were presented in [29]. 

**Figure 1:**
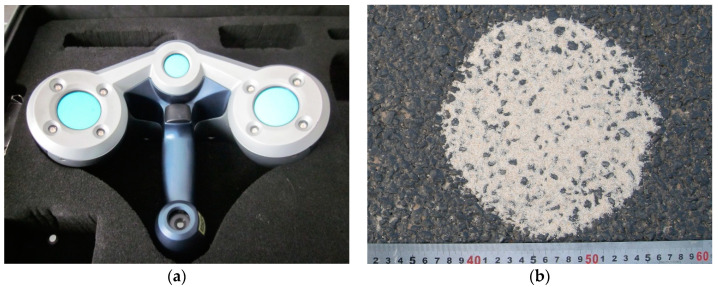

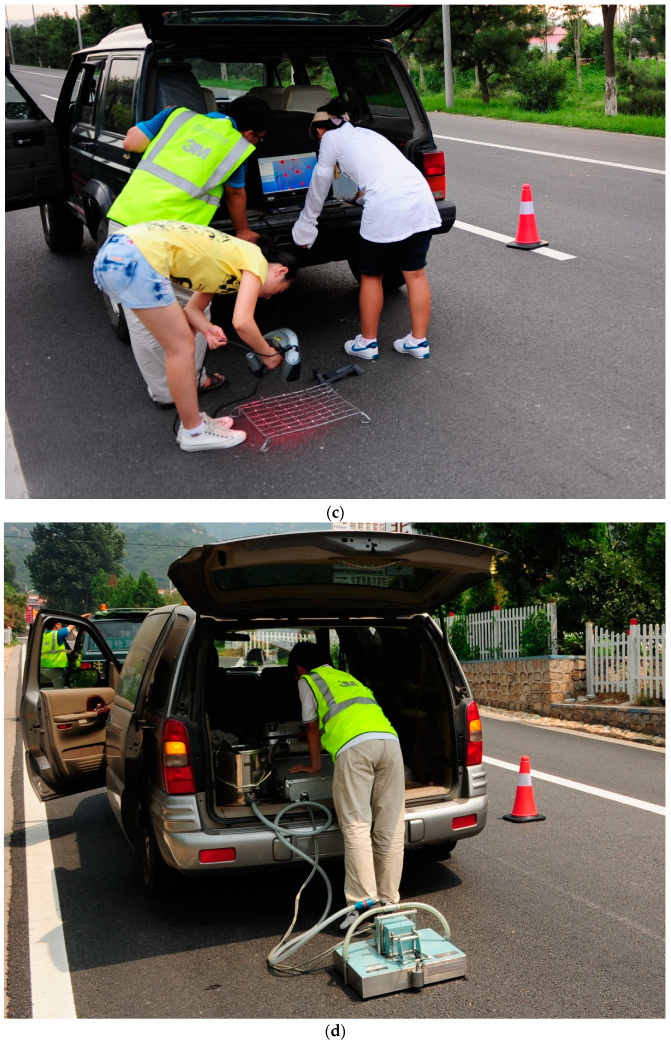
Field tests: (**a**) the 3-D scanner; (**b**) sand patch test for the mean texture depth (MTD); (**c**) Scanning test; and (**d**) dynamic friction tester (DFT) test.

**Figure 2:**
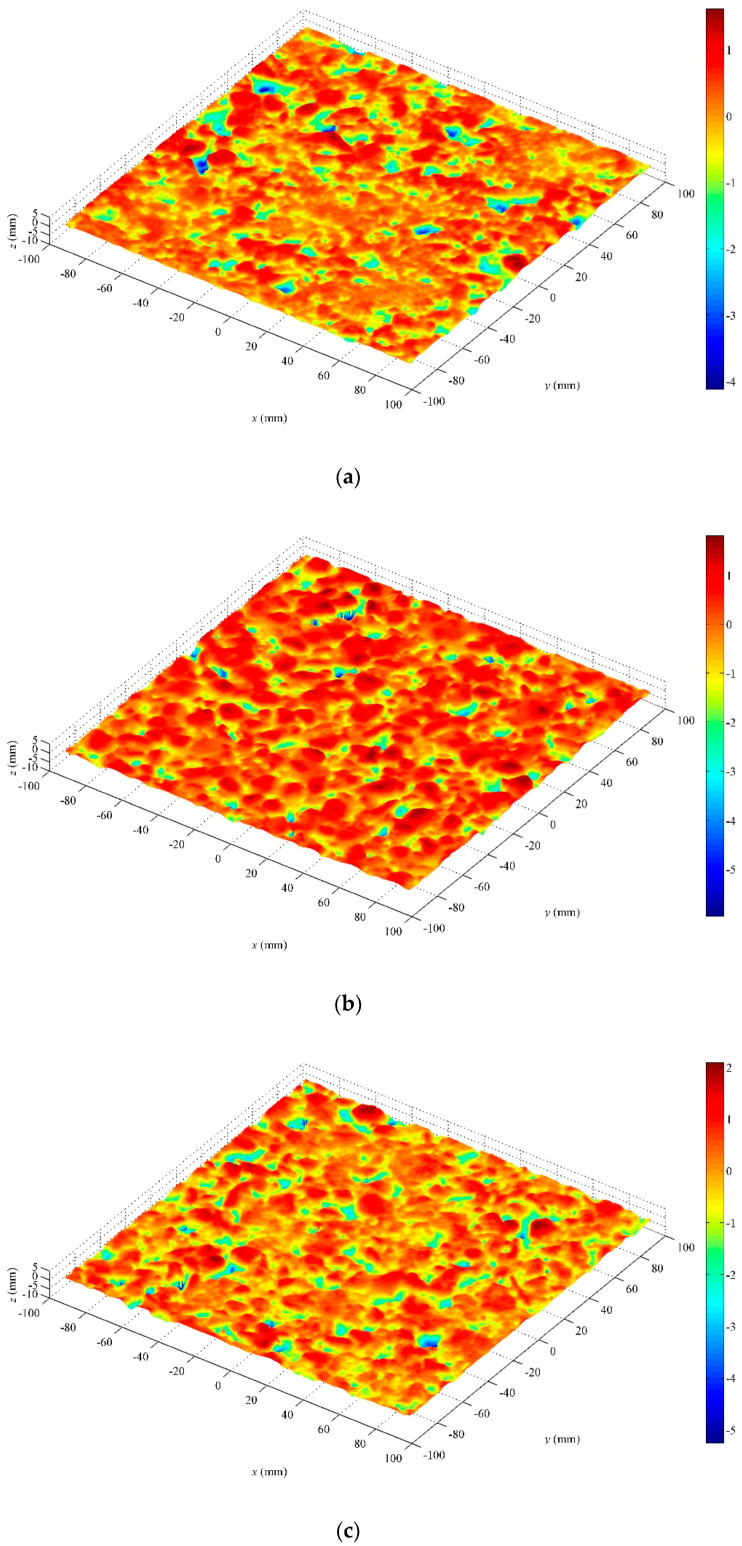

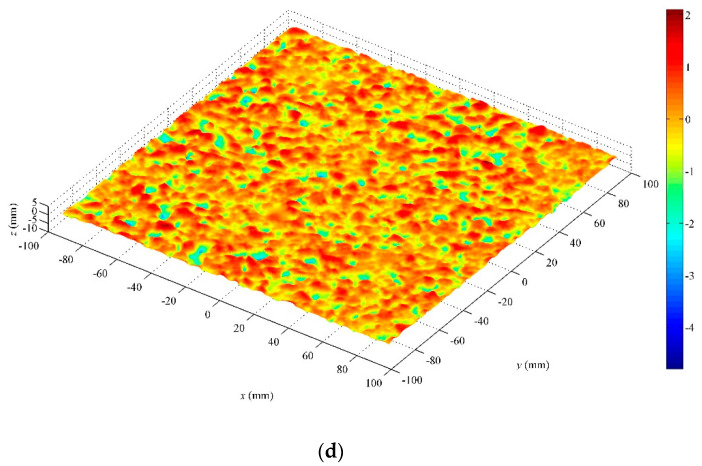
Results of a typical 3-D macrotexture: (**a**) dense asphalt concrete (DAC); (**b**) stone matrix asphalt (SMA); (**c**) rubber asphalt concrete (RAC); and (**d**) ultra-thin wearing course (UTWC).

**Figure 3:**
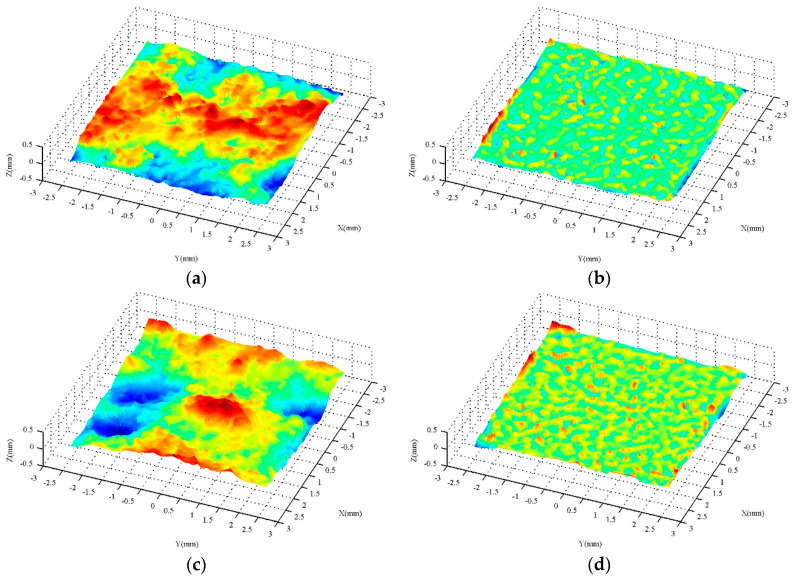
Results of a typical 3-D microtexture: (**a**) DAC, measured; (**b**) DAC, filtered; (**c**) SMA, measured; and (**d**) SMA, filtered.

A commercial hand-held 3-D laser scanner (Creaform Inc., Lévis, QC, Canada), based on the laser triangulation technique, is used to collect the three-dimensional macro and micro textures of the pavement surface. The scanner consists of three charge-coupled devices (CCDs) and a cross laser [24]. By collecting the coordinates of a series of points on the surface of the object, the 3-D image of the surface can be obtained. The minimum sampling point spacing is 0.05 mm, and the measuring accuracy is 0.04 mm. The test results can be outputted to a variety of standard 3-D image file formats, such as stl., iges., etc. For more detailed information, refer to [7]. Figure 1 shows the field test photos. For the macrotexture, in the first two tests, the sampling size was 90 mm × 90 mm, and in the last five tests, the sampling size was 190 mm × 190 mm. All macrotexture scanners use a sampling interval of 0.4 mm to edit the 3-D images obtained by scanning. First, the commercial 3-D image software Geomagic Studio (3D Systems, Inc., Research Triangle Park, NC, USA) is used to edit the images, and then the data is transformed into ordered point clouds with an equal spacing distribution in two horizontal directions through the simulation scanning tool. Furthermore, the Fast Fourier Transform is used to filter the part whose wavelength exceeds 50 mm. According to [4], these components are beyond the scope of the macrotexture. The ordered point cloud data with a sampling interval of 0.5 mm in the x and y directions are finally obtained, and used for the macrotexture analysis. The detailed information was described in detail in [7]. For the microtexture, the test method was the same as for the macrotexture, except that a sampling interval of 0.05 mm was used. Based on the scanned three-dimensional data, the filtering process is carried out according to the frequency and wavelength range of the microtexture [4]. Finally, the ordered point cloud data with a size of 5 mm × 5 mm and a sampling interval of 0.05 mm in the *x* and *y* directions are obtained for the analysis of the microtexture. One macrotexture and two microtextures are collected at each test point. Figure 2 is the result of typical macrotexture testing after filtering, and Figure 3 is the result of a typical microtexture by measuring and after filtering. For the macrotexture, since only the part whose wavelength is larger than 50 mm is filtered out, this wavelength value is several times the size of the particle exposed on the pavement surface, and the filtered image has not intuitively changed. For the microtexture, because it is necessary to filter out the part whose wavelength is more than 0.5 mm, which is much smaller than the size of the particle exposed on the pavement surface, the filtered microtexture loses the true morphology characteristics of the pavement surface.

## 3. Characterizing Surface Texture of Asphalt Pavement Using Entropy Theory

Denote a grey level image as *I*= {*G*(*x*, *y*), *x* = 1, 2, …, *N_x_*, *y* = 1, 2, …, *N_y_*}, where *G*(*x*, *y*) is the grey level at (*x*, *y*), and *N_x_* and *N_y_* are the pixel numbers along the *x* and *y* directions respectively. Following this, the probability of grey level *i* is

(1) $$p_{i}=\frac{\sum_{x=1}^{N_{x}}\sum_{y=1}^{N_{y}}\delta \left(G\left(x,y\right),i\right)}{N_{x}\times N_{y}}$$

where *δ*(*i*,*j*) is the Kronecker delta function.

(2) $$\delta (i,j)=\{\begin{array}{cc} 1 & i=j\\ 0 & i\neq j \end{array}$$

If an image has the maximum grey level of *N_g_*, the entropy (*E*) of the image can be defined as [34]

(3) $$E=\sum_{i=1}^{N_{g}}p_{i}\;\log_{2}\;\left(\frac{1}{p_{i}}\right)$$

**Figure 4:**
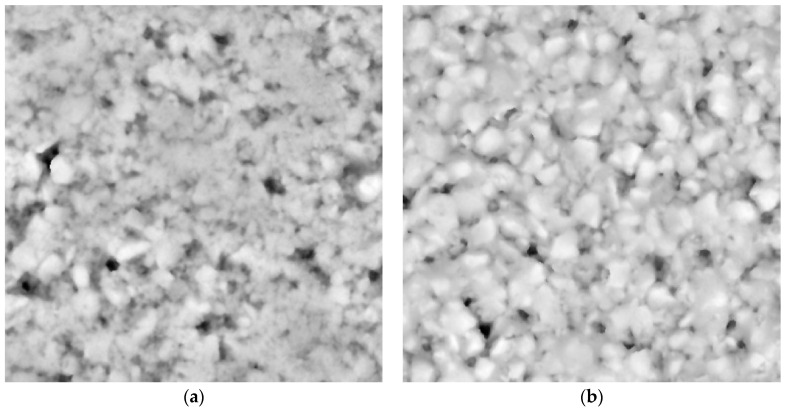

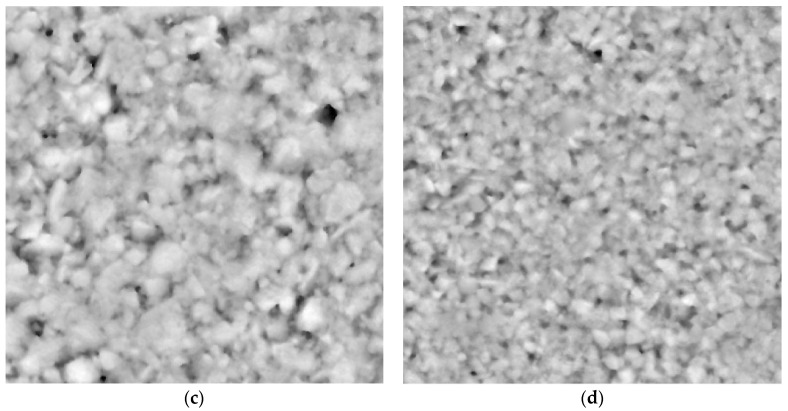
Grey-level images corresponding to the macrotexture shown in Figure 2: (**a**) DAC; (**b**) SMA; (**c**) RAC; and (**d**) UTWC.

**Figure 5:**
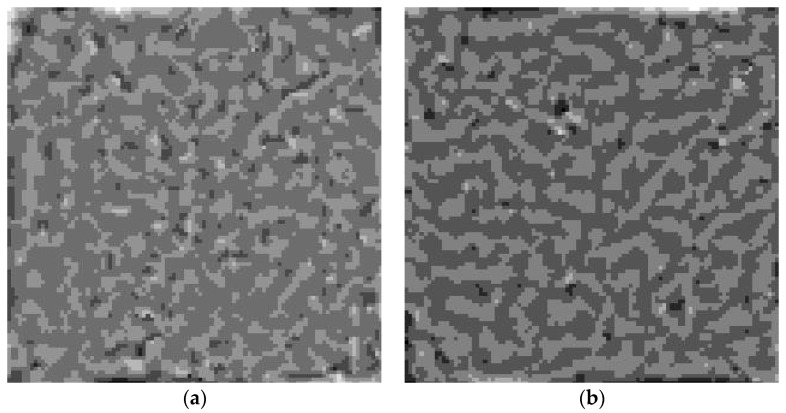
Grey-level images corresponding to the microtexture shown in Figure 3: (**a**) SMA and (**b**) DAC.

The 3-D texture measurements should be converted into grey-level images so that they can be characterized by entropy. First, the height range of a given 3-D texture measurement is divided into sections using a given interval. Following this, a corresponding grey-level image is obtained through mapping each height section onto a grey level. Reference [24] describes the conversion techniques in detail. Figure 4 and Figure 5 depict the grey-level images corresponding to the 3-D measurements in Figure 2 and Figure 3, respectively. Finally, the Entropy *E* can be calculated for each 3-D texture measurement in accordance with Equation (3).

**Figure 6:**
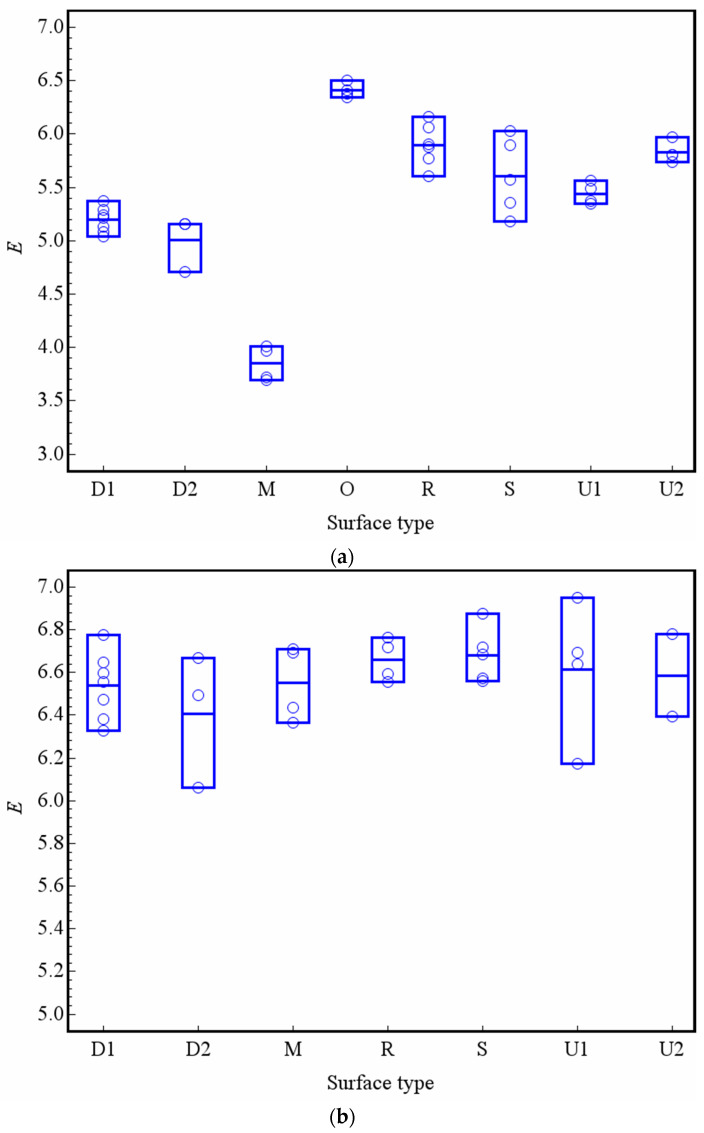
Distribution of entropy of the macrotexture and microtexture: (**a**) Macrotexture and (**b**) Microtexture.

Figure 6 presents the entropy distribution of the macrotexture and microtexture of different types of pavements, where D1 and D2 represents the DAC pavement, M represents the MS pavement, O represents the OGFC pavement, R represents the RAC pavement, S represents the SMA pavement, and U1 and U2 represent the UTWC pavements constructed over different years. The detailed information of various pavement parameters is referred to in [29]. As shown in Figure 6a, there are significant differences in the entropy of the macrotexture of different types of pavement surfaces. Among them, the entropy of MS pavement is the smallest and that of OGFC pavement is the largest. It is noted that there is a clear distinction between U1 and U2, in which U1 is the pavement opened in September, 2009, and U2 is the pavement opened in September, 2010; this indicates that entropy can be used to describe the macrotexture decay of the pavement surface. Figure 6a has the same distribution trends as those of previous research [24], indicating that the use of entropy is reasonable and accurate. For the microtexture, the range of the entropy distribution is narrow (Figure 6b), and the difference between different pavements is not very obvious. This may be because the microtexture is mainly affected by mineral aggregates and is less affected by mixture gradation.

## 4. Characterization of Macrotexture Degradation with Entropy

### 4.1. Degradation of Macrotexture Entropy

**Figure 7:**
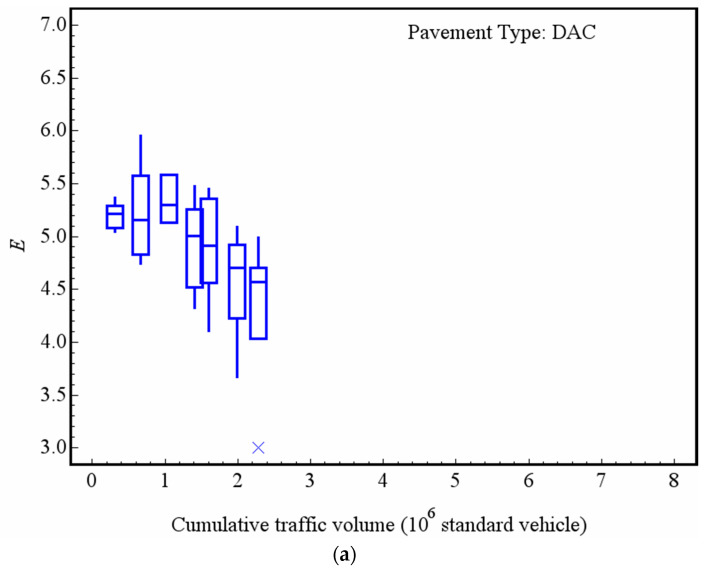

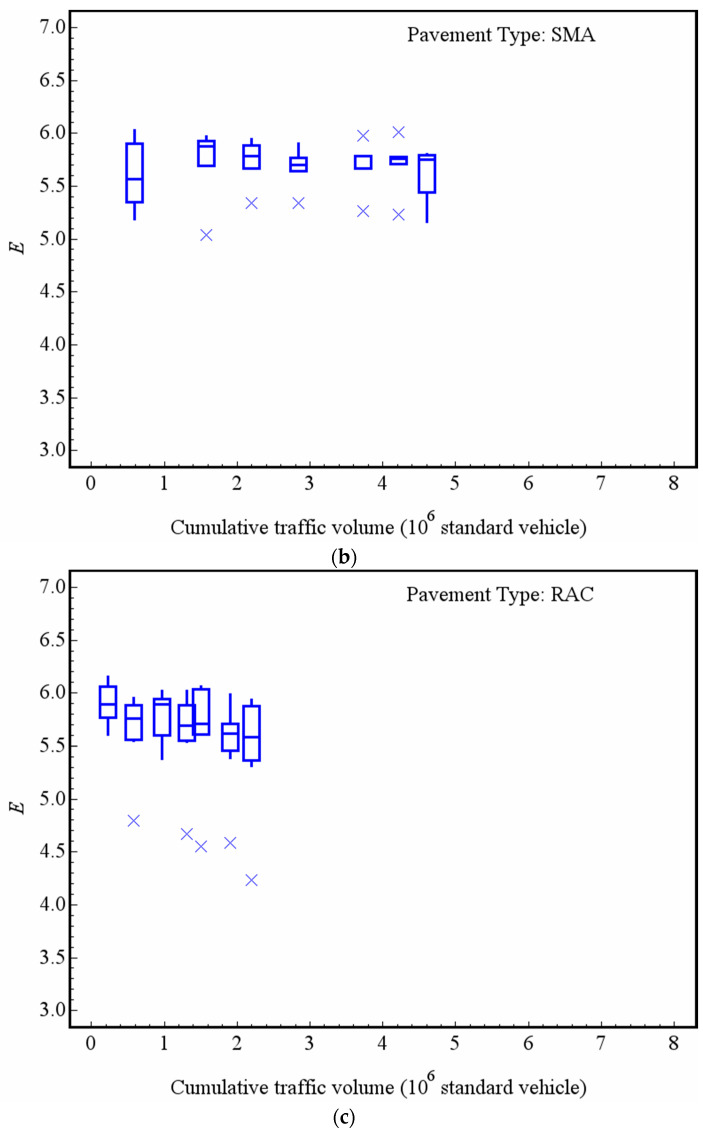

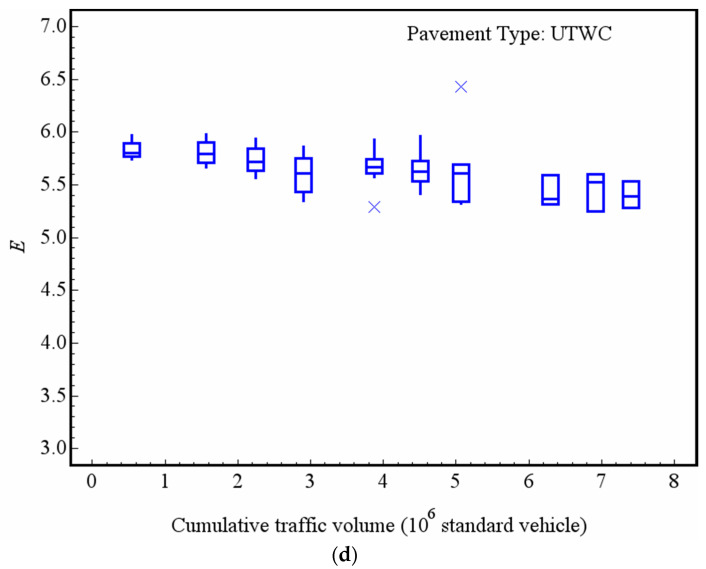
Change of the macrotexture entropy with the traffic volume: (**a**) DAC; (**b**) SMA; (**c**) RAC; and (**d**) UTWC.

In order to analyze the decay of the macrotexture of the asphalt pavement by the entropy value, the experimental data is grouped according to the type of pavement and cumulative traffic volume of service. Figure 7 presents the changes of entropy of the macrotextures of DAC, SMA, RAC and UTWC pavement surfaces with cumulative traffic volume by box-and-whisker plots, in which the mark inside the box is the median, the lower and the upper edges of the box are the 1st and 3rd quartiles, respectively, and the “x” are the outliers. Before analyzing the decay trend, it is necessary to note that the aggregate types used in the four types of pavement are not identical. DAC and RAC pavements used one type of aggregate, while SMA and UTWC pavement used another type. In the early service stage of roads, the entropy of the RAC surface macrotexture is the largest amongst the four types of pavement, with an average value of 5.90 (after a 0.22 × 10^6^ standard vehicle passes), followed by the UTWC and SMA pavements, with an average value of 5.83 (after a 0.54 × 10^6^ standard vehicle passes) and 5.61 (after a 0.59 × 10^6^ standard passes), respectively. The entropy of the surface macrotexture of DAC is the smallest, with an average value of 5.20 (after a 0.31 × 10^6^ standard vehicle passes). Despite some fluctuations in data, for the DAC, RAC and UTWC pavements, it is clear that the entropy of the macrotexture of the pavement surface decreases gradually with the increase of the cumulative traffic volume. The DAC pavement decay is the most obvious. After a 2.29 × 10^6^ standard vehicle passes, the average entropy of DAC’s macrotexture decays to 4.34, and the average entropy of DAC’s macrotexture decays to 8.37% for every 1 × 10^6^ standard vehicle passing. After a 2.20 × 10^6^ standard vehicle passes, the average entropy of RAC’s macrotexture decays to 5.48, with an average decay rate of 3.6% for every 1 × 10^6^ standard vehicle passing. After a 7.40 × 10^6^ standard vehicle passes UTWC, the average entropy of the macrotexture decays to 5.40, and the average decay rate is 1.07% for every 1 × 10^6^ standard vehicle passing, respectively. The entropy of the macrotexture of the SMA pavement surface does not obviously decay. After a 4.61 × 10^6^ standard vehicle passes, the average entropy of the macrotexture is still 5.59, which is basically consistent with the mean value of a 0.59 × 10^6^ standard vehicle passing. The analysis using entropy theory obtains similar results compared with our previous research [29]. Pavements with different gradations will have different sizes of aggregates exposed on the surfaces. Due to the difference of wear performance of different size particles, there are different decay trends in the macrotexture of different pavements. On the other hand, the difference of aggregate types should also be noticed.

### 4.2. Changing Trends of Entropy in Macrotexture

In order to quantitatively depict the decay trend of the macrotexture of the pavement surfaces, a logarithmic model (as shown in Equation (4)) is used for a regression analysis of the change trend of the average entropy with the cumulative traffic volume, corresponding to the different cumulative traffic volumes of each pavement. Table 1 gives the least squares analysis results of four kinds of road regression analyses. Table 2 lists the mean square errors (MSEs) and R-squares of regression analyses of four kinds of pavements. The regression parameters of the model are listed in Table 3, as [29].

(4) E=a×ln(traf)+b

where *traf* is the cumulative traffic volume, and *a* and *b* are the fitting coefficients.

**Figure 8:**
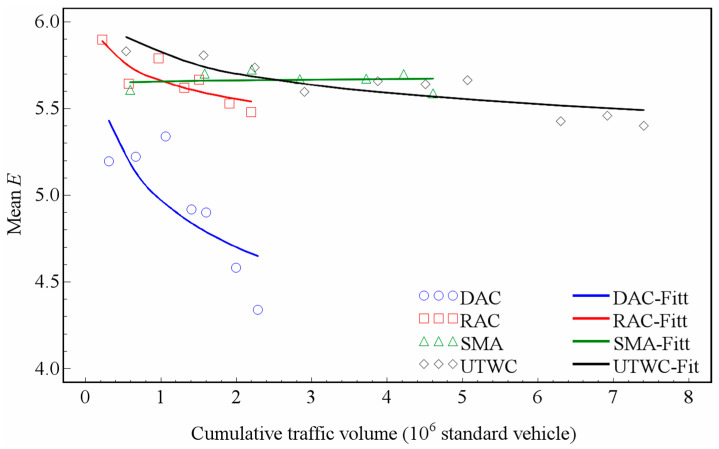
Changing trends of the macrotexture entropy.

As shown in Table 1, for the DAC, RAC, and UTWC pavements, the *p*-value is below 0.05, indicating that the logarithmic model is significant for these three types of pavement. For the SMA pavement, the *p*-value is 0.7509, much higher than 0.05, indicating that the logarithmic model is not significant for the SMA pavement, which is mainly due to the fact that the entropy of the macrotexture of the SMA pavement has not decayed significantly over more than two years of observation. Although the logarithmic model is not significant for the SMA pavement, Table 2 shows that the MSE fitted by the model is only 0.0030. Figure 8 presents the changes of the average entropy of four types of pavement macrotextures fitted by the logarithmic model.

According to Equation (4), the coefficient *a* described the decay rate. The smaller the value of *a*, the faster the decay rate. As mentioned above, the DAC and RAC pavements have one same aggregate type, and the SMA and UTWC pavements have another type. Because the aggregate type has a potential impact on the pavement wear, the decay of the macrotexture entropy of the pavement surface should take the difference between different aggregate types into account. From Table 3 and Figure 8, it can be seen that the macrotexture of the DAC pavement decreases fastest. The RAC and UTWC pavements have a similar macrotexture decay trend. The SMA pavement maintains a stable entropy after 4.61 × 10^6^ standard vehicle passes, which should be attributed to the specific gradation of SMA. Note that the trends using the mean entropy are very similar to those from the previous analysis [29], which validates our research accuracy.

### 4.3. Relationship of Macrotexture Entropy and MTD with DFT60

In the field tests from November 2010 to November 2012, the mean texture depth (MTD) was tested by a sand patch method at each test point, and the friction performance of the pavement was tested by a dynamic friction tester (Nippo Sangyo Co., Ltd., Tokyo, Japan), which is described in detail in [29]. In order to investigate the potential advantages of the entropy theory in describing the texture characteristics of asphalt pavement surfaces, based on the mean values of entropy, MTD and DFT60 for each pavement with different cumulative traffic volumes, the correlation among *E*, MTD and DFT60 are analyzed. The Pearson correlation coefficients are listed in Table 4.

**Figure 9:**
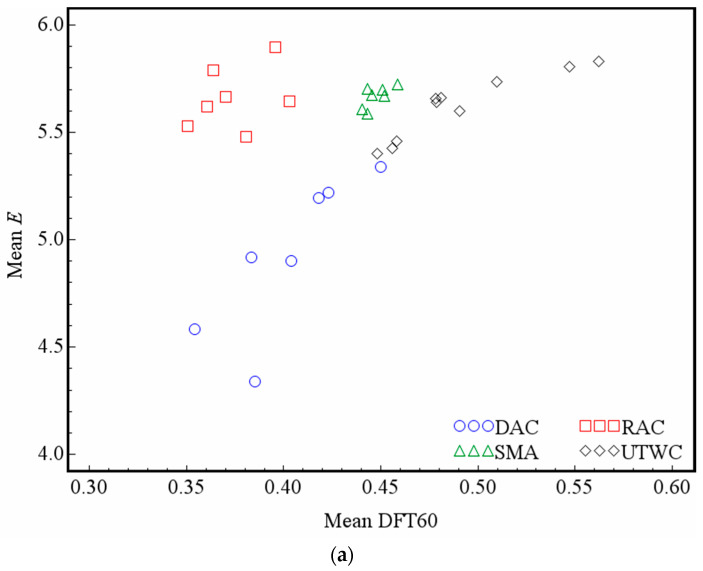

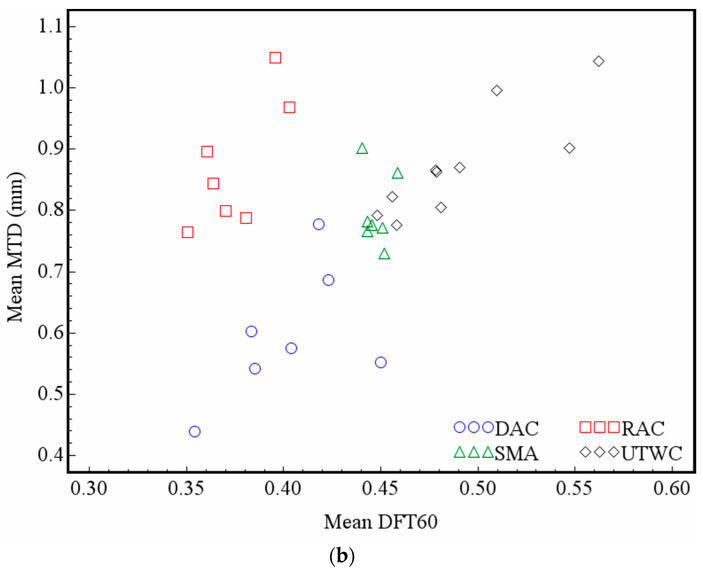
Scatter plots of DFT60 against *E* and MTD: (**a**) DFT60-*E* and (**b**) DFT60-MTD.

It can be seen from Table 4 that for the DAC, RAC, and UTWC pavements, there is a certain correlation between entropy and MTD. However, the Pearson correlation coefficients are below 0.8, and there is no significant correlation between the entropy and MTD of the SMA pavement. This shows that entropy describes some features of the macrotexture of the pavement surface, which the MTD indexes fail to describe. Comparing the correlations between entropy, MTD and DFT60, it is found that the Pearson correlation coefficients of entropy and DFT60 are significantly higher than those of MTD and DFT60 except for RAC. For the DAC, SMA and UTWC pavements, the correlation coefficients of entropy and DFT60 are 0.2960, 0.6401 and 0.0695 higher than the Pearson correlation coefficients of MTD and DFT60, respectively. This shows that entropy has more advantages than MTD in describing the impact of macrotexture on the anti-skid performance of asphalt pavement. Figure 9 plots DFT60 against the entropy *E* and MTD of different types of asphalt pavements, which could more intuitively reflect the advantages of *E*.

## 5. Characterization of Microtexture Degradation with Entropy

### 5.1. Degradation of Entropy of Microtexture

**Figure 10:**
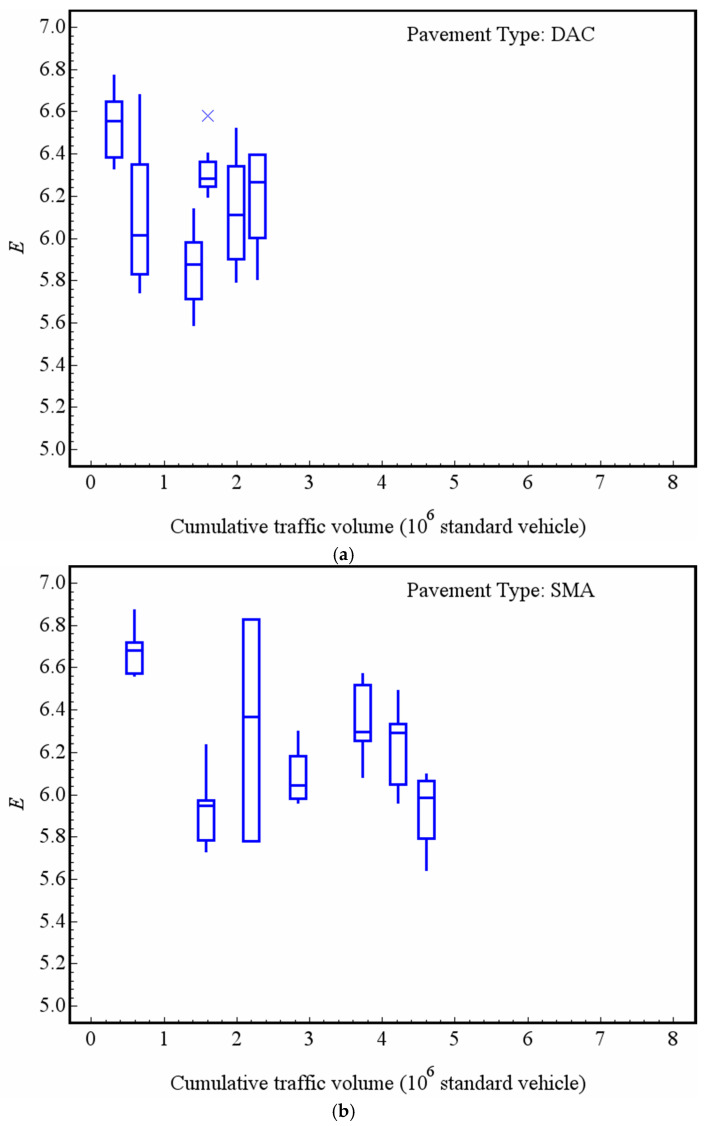

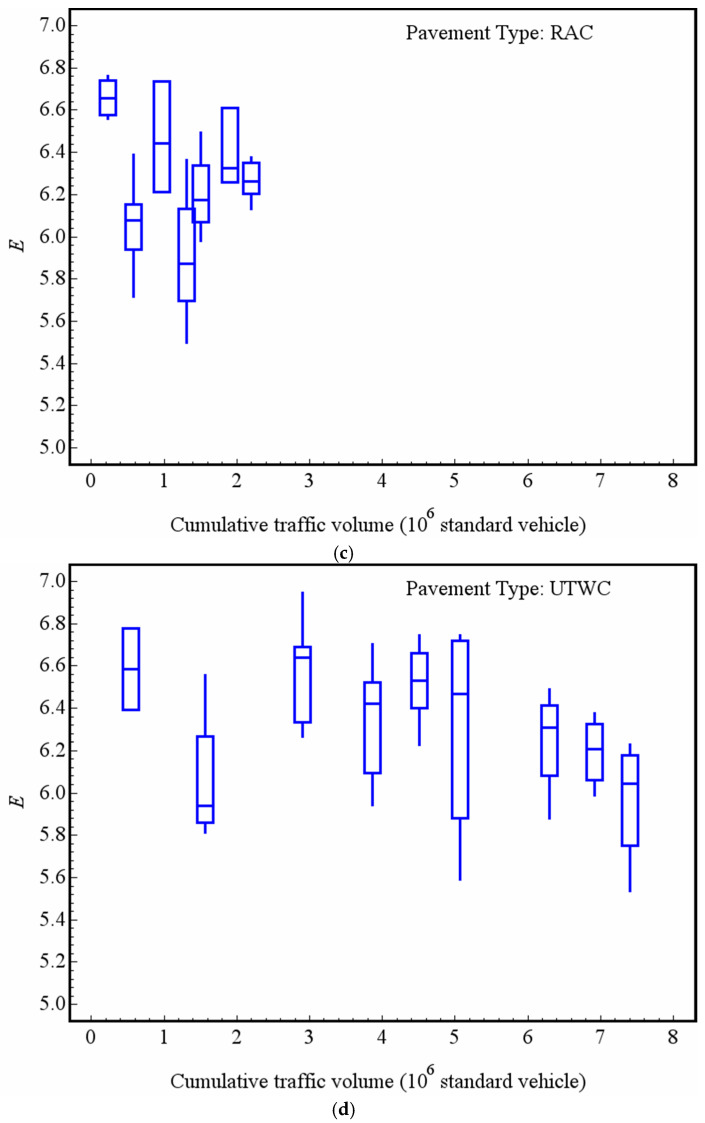
Change of the microtexture entropy with the traffic volume: (**a**) DAC; (**b**) SMA; (**c**) RAC; and (**d**) UTWC.

In the field test, two microtextures were collected at each test point, and the mean value of the two microtextures’ entropy is used as the evaluation basis for the feature evaluation. Similar to the macrotexture, the experimental data is grouped according to the cumulative traffic volume of different pavement types and services. Figure 10 gives the variation of the microtexture entropy of the DAC, SMA, RAC and UTWC pavement surfaces with the cumulative traffic volume by box-and-whisker plots, where the mark inside the box is the median, the lower and the upper edges of the box are the 1st and 3rd quartiles, respectively, and the “x” are the outliers. Note that the aggregate types used in the four types of pavement are not identical. The DAC and RAC pavements used one same type of aggregate, while the SMA and UTWC pavements used another. In the early service stage of roads, the average values of DAC, SMA, RAC and UTWC are 6.54 (after a 0.31 × 10^6^ standard vehicle passes), 6.68 (after a 0.59 × 10^6^ standard vehicle passes), 6.66 (after a 0.22 × 10^6^ standard vehicle passes), and 6.59 (after a 0.54 × 10^6^ standard vehicle passes).

Because the decay of microtexture is mainly caused by the polishing of aggregate particles on the surface of pavements, the decay of microtexture is relatively slow, and the absolute value of entropy decay is much smaller than that of macrotexture. Nevertheless, Figure 10 could identify that the entropy of the four types of pavement surface microtextures gradually decreases with the increase of the traffic volume. After a 2.29 × 10^6^ standard vehicle passes, the average entropy of the DAC texture decays to 6.19, and the average decay rate is 2.71% for every 1 × 10^6^ standard vehicle passing. The average entropy of the microtexture of the RAC pavement decays to 6.27 after a 2.20 × 10^6^ standard vehicle passes, and the average decay rate is 2.97% for every 1 × 10^6^ standard vehicle passing. The average entropy of the SMA pavement texture decays to 5.93 when a 4.61 × 10^6^ standard vehicle passes, with an average decay rate of 2.80% for every 1 × 10^6^ standard vehicle passing. After a 7.40 × 10^6^ standard vehicle passed the UTWC pavement, the average entropy of the microtexture decays to 5.96, and the average decay rate is 1.37% for every 1 × 10^6^ standard vehicle passing. The microtexture decay behavior of different types of pavements is similar.

### 5.2. Changing Trends of Entropy of Microtexture

In order to quantitatively depict the decay trend of the microtexture of the pavement surface, based on the average value of entropy corresponding to the different cumulative traffic volume of each pavement, the power function model (as shown in Equation (5)) is used to analyze the trend of the average entropy with the cumulative traffic volume by comparing various models. Table 5 shows the least squares analysis results of four kinds of road regression analyses. Table 6 lists the mean square errors (MSEs) and R-squares of a regression model. The regression parameters of the model are listed in Table 7, as [29].

(5) $$E=a\times traf^{b}$$

where *traf* is the cumulative traffic volume, and *a* and *b* are the fitting coefficients.

**Figure 11:**
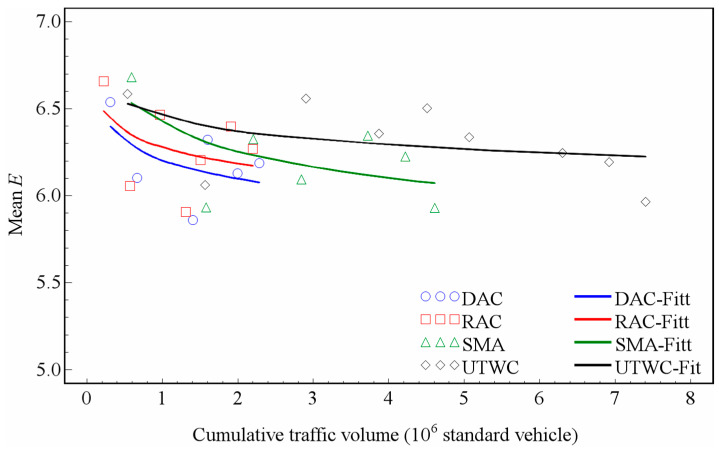
Changing trends of the microtexture entropy.

For the four types of pavements, all the *p*-values below are 0.0001. Table 5 shows that the R-square value is low, and the SMA pavement with the largest R-square value is only 0.3676, indicating that the regression model is not very ideal. This is mainly due to the small decay variation of the microtexture entropy and the fluctuation of the test data. Figure 11 presents the variation curves of the average entropy of the four types of pavement microtextures fitted by power model. Although the regression model is not perfect, the model can still be used for some simple analyses.

According to Equation (5), coefficient *b* describes the decay rate, and the larger the value *b*, the faster the decay rate. As mentioned above, the DAC and RAC pavements use one same aggregate type and the SMA and UTWC pavements use another type. This difference should be noticed when analyzing the difference of microtexture entropy between the different pavements. According to Table 7 and Figure 11, the SMA pavement surface texture decay rate is the fastest, followed by the DAC and RAC pavements, and the UTWC pavement decay rate is the slowest. The average decay of entropy is less than 2.7% for every 1 × 10^6^ standard vehicle passing, for all four types of pavements.

## 6. Discussion

According to the definition of pavement texture entropy in Equations (1) to (3), it can be seen that the possible range of entropy of the macro- or micro- textures of the pavement surfaces is [0, log_2_(*N_g_*)]. In a plane, if there is only one grey value, the *p_i_* of this grey value is 1, which leads to zero entropy. For the same texture with *N_g_*, when all grey values correspond to the same *p_i_*, the entropy reaches the maximum value [34]. The larger the *N_g_*, the greater the maximum value. For the pavement surface texture, the complexity of the texture increases with the increase of the entropy. Under the polishing action of the vehicle load, the surface of the pavement tends to be smooth, showing the entropy decreasing gradually. Compared with the MTD index, entropy contains more physical information. At the same time, the correlation between macrotexture entropy and DFT60 is significantly higher than for MTD. The entropy has obvious advantages over the traditional MTD index in the macrotexture evaluation of pavement anti-skid performance.

According to the test results in this paper, the difference of microtexture entropy between different pavements is not very significant, and the law of decreasing with traffic volume is not very significant. On one hand, the microtexture is mainly influenced by aggregate particles, and the difference of aggregates between different pavements is not prominent. On the other hand, the polishing process of the aggregate surface is relatively slow, and may require a larger load to show a significant decay trend.

It should be noted that the wavelength of microtextures is less than 0.5 mm according to the division of the texture scale, which requires a very small sampling interval to reflect the real microtexture. Some researchers used a 0.001 mm sampling interval to test and analyze microtextures in laboratory experiments [11]. However, in order to evaluate the microtexture of real pavements, it is necessary to sample the pavement by drilling holes, which causes great damage to the pavement surface. At present, there is no report on pavement field test equipment which can reach a 0.001 mm sampling distance. In this paper, a 3-D scanner with a 0.05 mm sampling distance is used to test and analyze the microtexture of the pavement field. Although the microtexture cannot be fully reflected in this study, the present research can, as an exploration, still provide guidance for future studies.

The valuation of pavement surface texture has important engineering application values. On the one hand, it can directly establish the relationship between the texture of road surfaces and anti-skid performance, which can be used to evaluate anti-skid performance. On the other hand, the pavement surface texture depends on the gradation of the asphalt mixture and the morphological characteristics of aggregates. The pavement surface texture is a bridge connecting the asphalt mixture design and the anti-skid performance of the pavement. The description of texture featured by traditional evaluation indexes provides an insufficient connection between texture evaluation and anti-skid performance, and thus the engineering value of texture evaluation has not been fully revealed, while the widely-used design method of anti-skid performance of asphalt pavement has not been formed. Our research results show that the use of entropy to describe the macrotexture of pavement surfaces contains more physical information, and indicates the relationship between macrotexture and anti-skid performance. On the one hand, with the accumulation of data by the popularized 3-D texture testing method in engineering practice, using entropy as a texture index can improve the evaluation of pavement anti-skid performance. On the other hand, it is feasible to use entropy as a texture index to connect the anti-skid performance of asphalt mixture and pavement and improve the design method of the anti-skid performance of asphalt pavement.

## 7. Conclusions

In this paper, based on the data of seven field tests on the surface texture and friction characteristics of various types of asphalt pavements over more than two years, the macro-/micro- texture characteristics and decay of 3-D asphalt pavement surfaces were studied using the theory of entropy. Through this research, the following conclusions can be drawn:

(1) The entropy distribution range of the 3-D macrotexture of asphalt pavements is wide, and there are significant differences among different gradation pavement types. There are significant differences in the entropy of the 3-D macrotextures of asphalt pavements with different mixture designs. The difference of 3-D microtextures is not very obvious. Furthermore, the distribution range of macrotexture entropy is wider than that of microtextures. The macrotexture of asphalt pavements is mainly affected by the gradation of mixture, while the microtexture is mainly affected by the surface morphology of aggregates.
(2) There are significant differences in the decay characteristics of 3-D macrotextures of asphalt pavements with different mixture types, which indicates that the decay characteristics of the macrotexture of asphalt pavement surfaces could be significantly improved by choosing appropriate mixture types and optimizing the design.
(3) Compared with the traditional macrotexture parameter MTD, entropy contains more physical information and a better correlation with the pavement anti-skid performance index. It has significant advantages in describing the relationship between macrotexture characteristics and anti-skid performances of asphalt pavements.
(4) This paper attempts to collect the 3-D microtexture of pavement surfaces with a 0.05 mm sampling interval. The decay law of the 3-D microtexture of different types of asphalt pavements is not very significant; this may require a longer observation time and more innovative methods to obtain more detailed microtextures for further studies.

## Figures and Tables

**Table 1:** Least-squares analysis of the model for the mean entropy of the macrotextures. Dense asphalt concrete (DAC); Stone matrix asphalt (SMA); Rubber asphalt concrete (RAC); and Ultra-thin wearing course (UTWC).

Surface Type	Model Sum of Squares	Error Sum of Squares	Corrected Total Sum of Squares	F Value	P > F
DAC	0.4518	0.3416	0.7935	6.61	0.0499
RAC	0.0893	0.0353	0.1246	12.63	0.0163
SMA	0.000343	0.0152	0.0156	0.11	0.7509
UTWC	0.1531	0.0548	0.208	22.35	0.0015

**Table 2:** The mean square errors (MSEs) and R-squares of the regression model for the mean entropy of the macrotextures.

DAC		RAC		SMA		UTWC	
MSE	R2	MSE	R2	MSE	R2	MSE	R2
0.0683	0.5695	0.0071	0.7167	0.0030	0.0256	0.0068	0.7365

**Table 3:** Fitting coefficients of the model for the mean entropy of macrotexture.

Surface Type	DAC	RAC	SMA	UTWC
a	−0.3929	−0.1525	0.0105	−0.1609
b	4.9734	5.6611	5.6569	5.8139

**Table 4:** Pearson correlation coefficients between the Mean E, Mean texture depth (MTD), and Mean DFT60.

Surface Type	DAC	RAC	SMA	UTWC
Mean E VS Mean MTD	0.6041	0.7139	−0.0932	0.7997
Mean E VS Mean DFT60	0.8283	0.3407	0.7036	0.9169
Mean MTD VS Mean DFT60	0.5323	0.7298	−0.0635	0.8474

**Table 5:** Least-squares analysis of the model for the mean entropy of the macrotextures.

Surface Type	Model Sum of Squares	Error Sum of Squares	Corrected Total Sum of Squares	F Value	P > F
DAC	229.9	0.1848	0.2590	2487.7	<0.0001
RAC	276.1	0.3141	0.3848	2197.2	<0.0001
SMA	270.9	0.267	0.4222	2536.0	<0.0001
UTWC	358.6	0.2981	0.3765	4210.4	<0.0001

**Table 6:** The MSEs and R-squares of the regression model for the mean entropy of the microtextures.

DAC		RAC		SMA		UTWC	
MSE	R2	MSE	R2	MSE	R2	MSE	R2
0.0462	0.2864	0.0628	0.1836	0.0534	0.3676	0.0426	0.2082

**Table 7:** Fitting coefficients of the model for the mean entropy of microtextures.

Surface Type	DAC	RAC	SMA	UTWC
a	6.2081	6.279	6.412	6.4559
b	−0.0257	−0.0216	−0.0357	−0.0183
